# Supplementary figures and images for: Estimating fine age structure and time trends in human contact patterns from coarse contact data: The Bayesian rate consistency model
Source: PLoS Comput Biol. 2023 Jun 5;19(6):e1011191. doi: 10.1371/journal.pcbi.1011191 (PMC10270591; doi:10.1371/journal.pcbi.1011191)

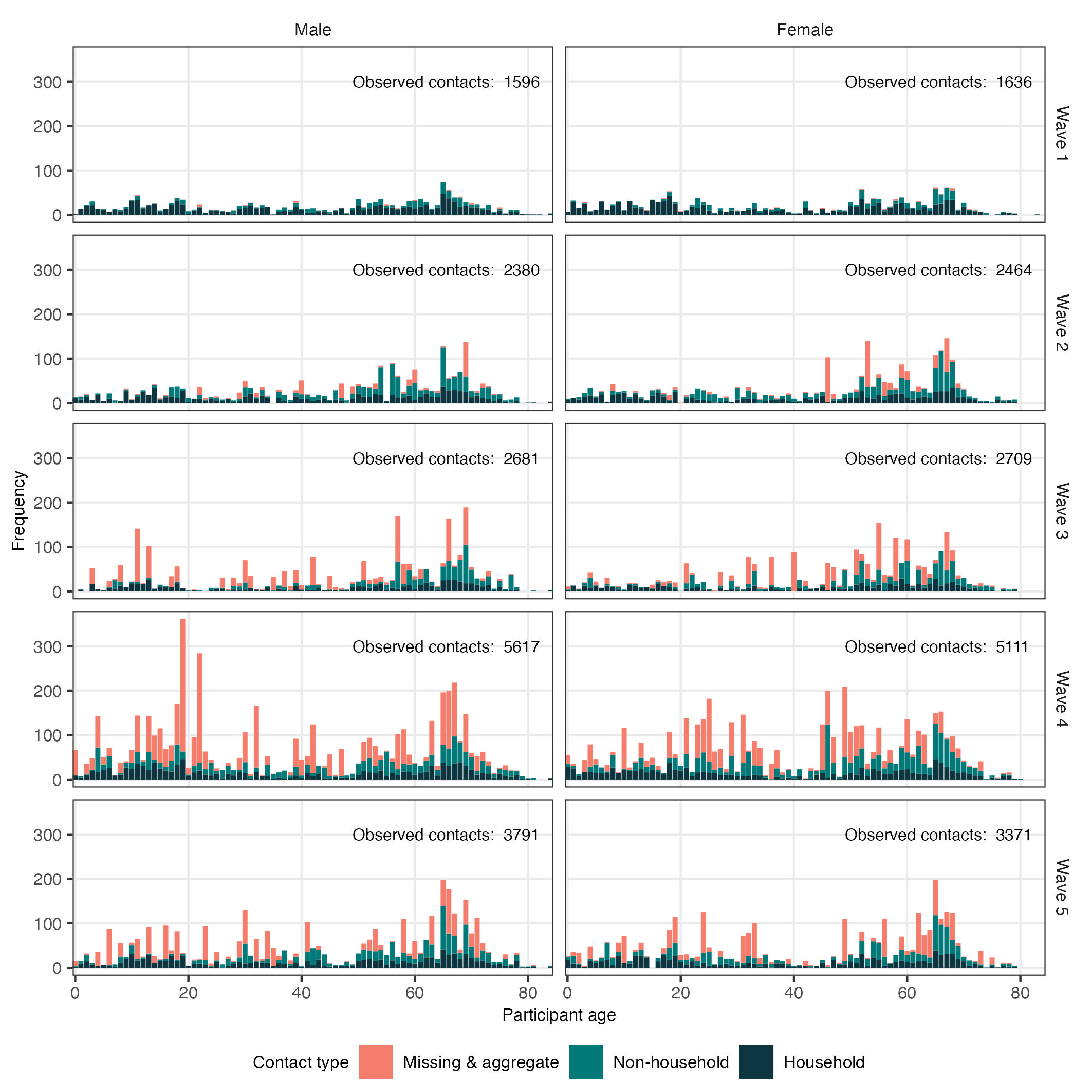

Supplement: S1 Fig — Pink bars represent missing & aggregate contacts, light blue bars represent non-household contacts, and dark blue bars represent household contacts. Missing & aggregate contacts were truncated at 60 (90th percentile in the primary data) to remove the effects of extreme outliers. (JPEG) [file pcbi.1011191.s001.jpeg]

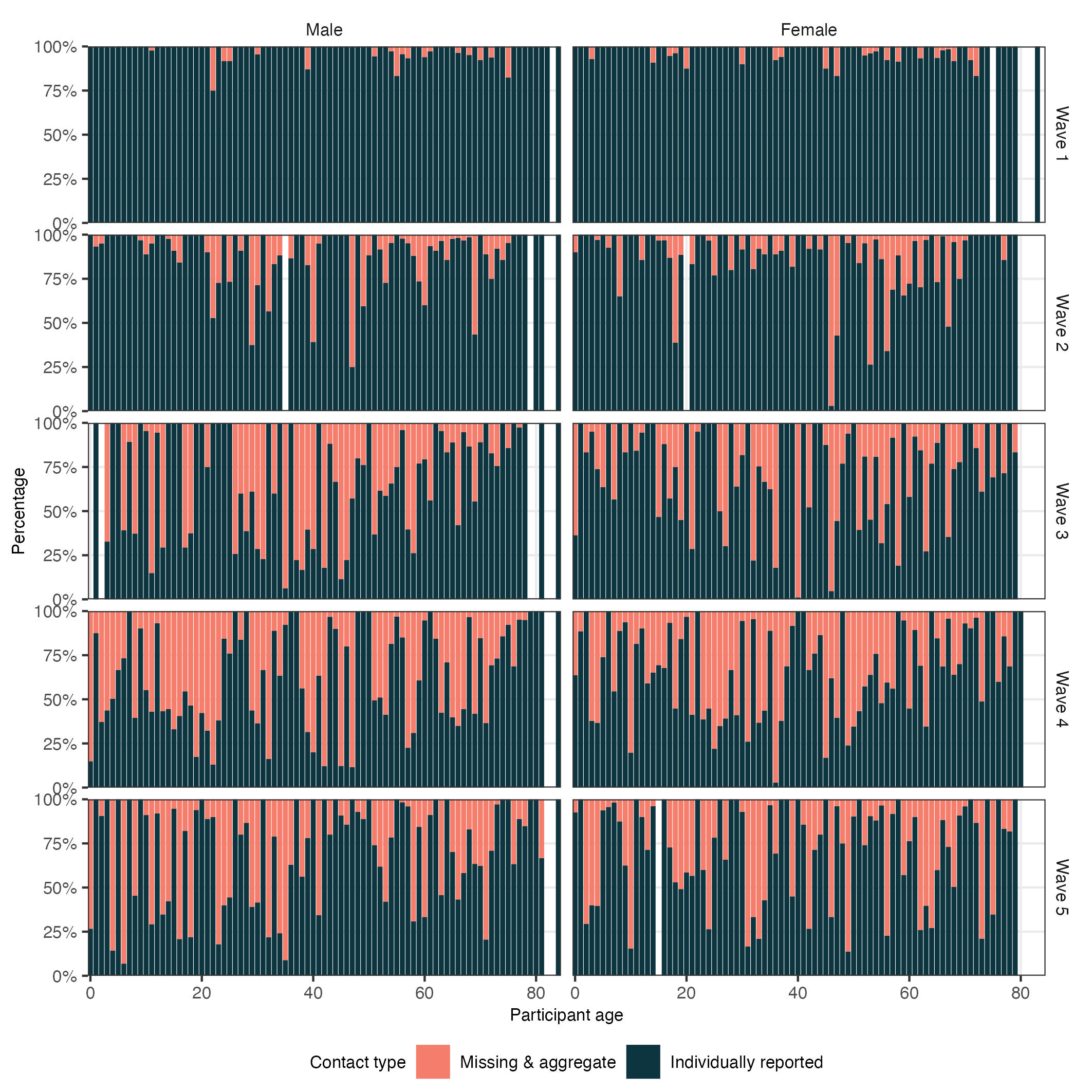

Supplement: S2 Fig — Dark blue bars represent individually reported contacts for whom the age of the contact was specified by age bands. Pink bars represent reported contacts that were either reported in aggregate or individually without detail on the age or gender of the contact. (JPEG) [file pcbi.1011191.s002.jpeg]

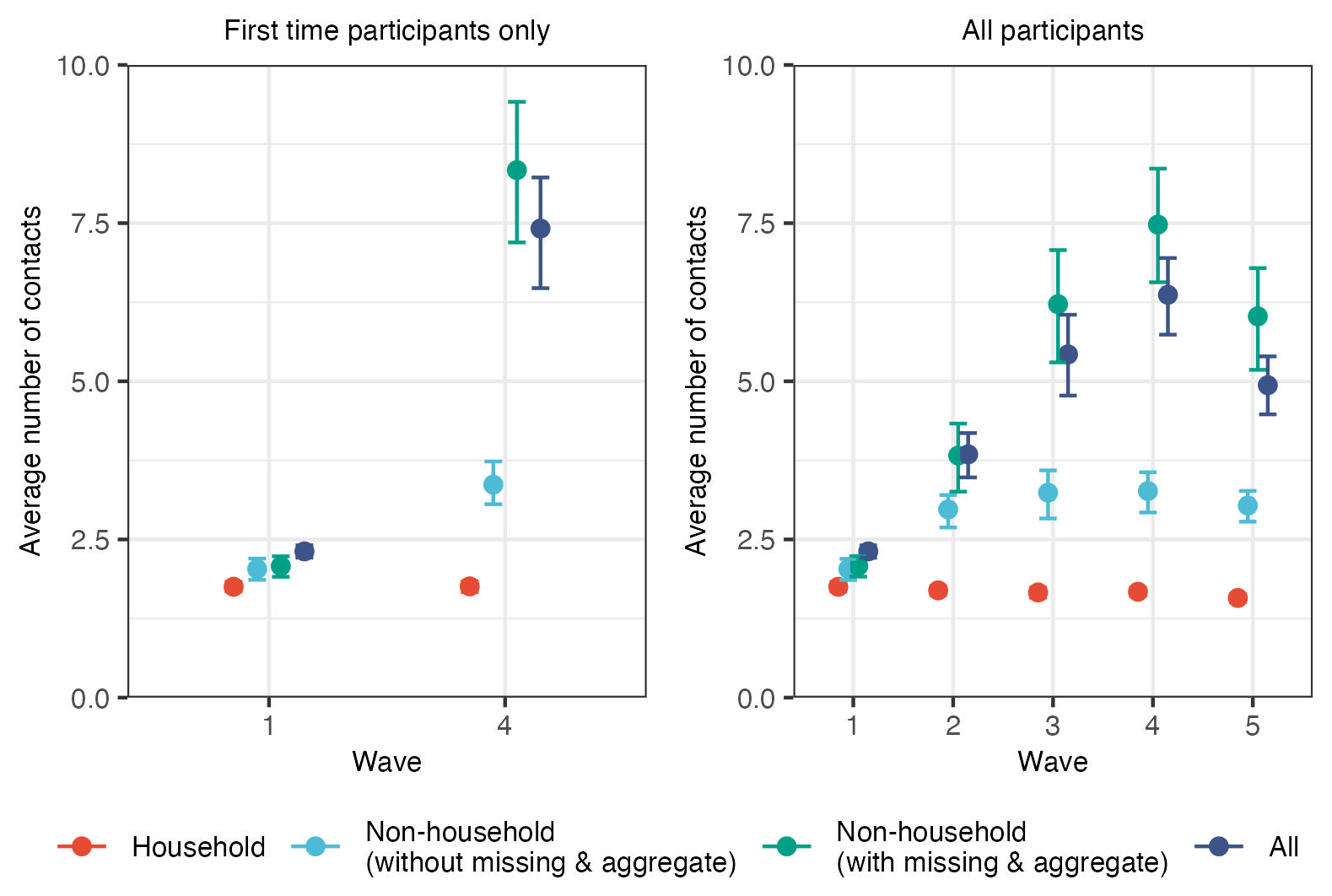

Supplement: S3 Fig — Red, light blue, green, and dark blue points represent the average number of household, non-household excluding aggregated & missing contacts, non-household including aggregated & missing contacts, and all contacts, respectively. Error bars represent 95% bootstrap confidence intervals. (JPEG) [file pcbi.1011191.s003.jpeg]

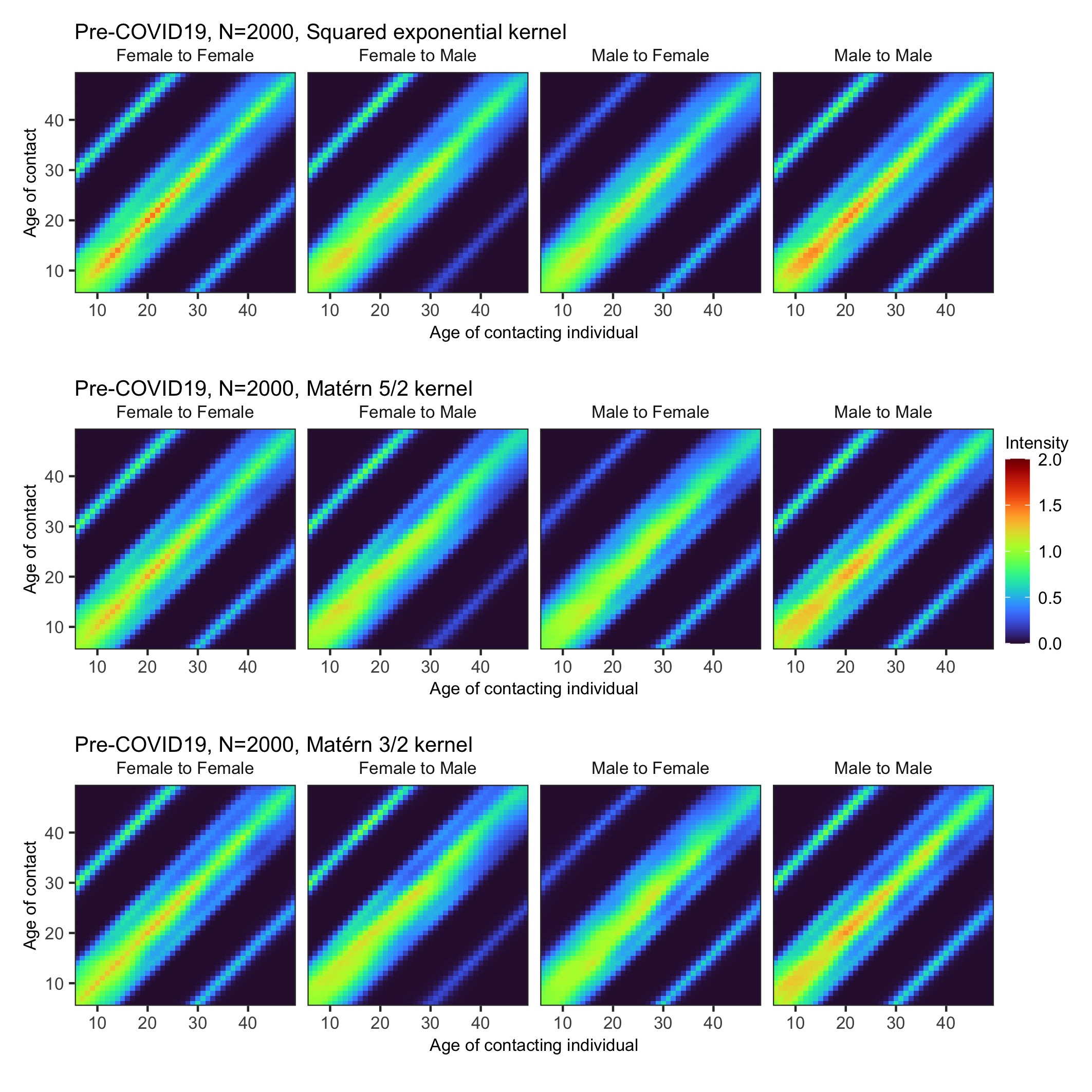

Supplement: S4 Fig — From top to bottom: results for the squared exponential kernel, results for the Matérn 52 kernel, and results for the Matérn 32 kernel. All experiments were run with HSGP using the difference-in-age parameterisation models with M1 = 40 (Number of eigenfunctions on the difference-in-age dimension) and M2 = 20 (Number of eigenfunctions on the contacts’ age dimension). The sample size was fixed at N = 2000. (JPEG) [file pcbi.1011191.s004.jpeg]

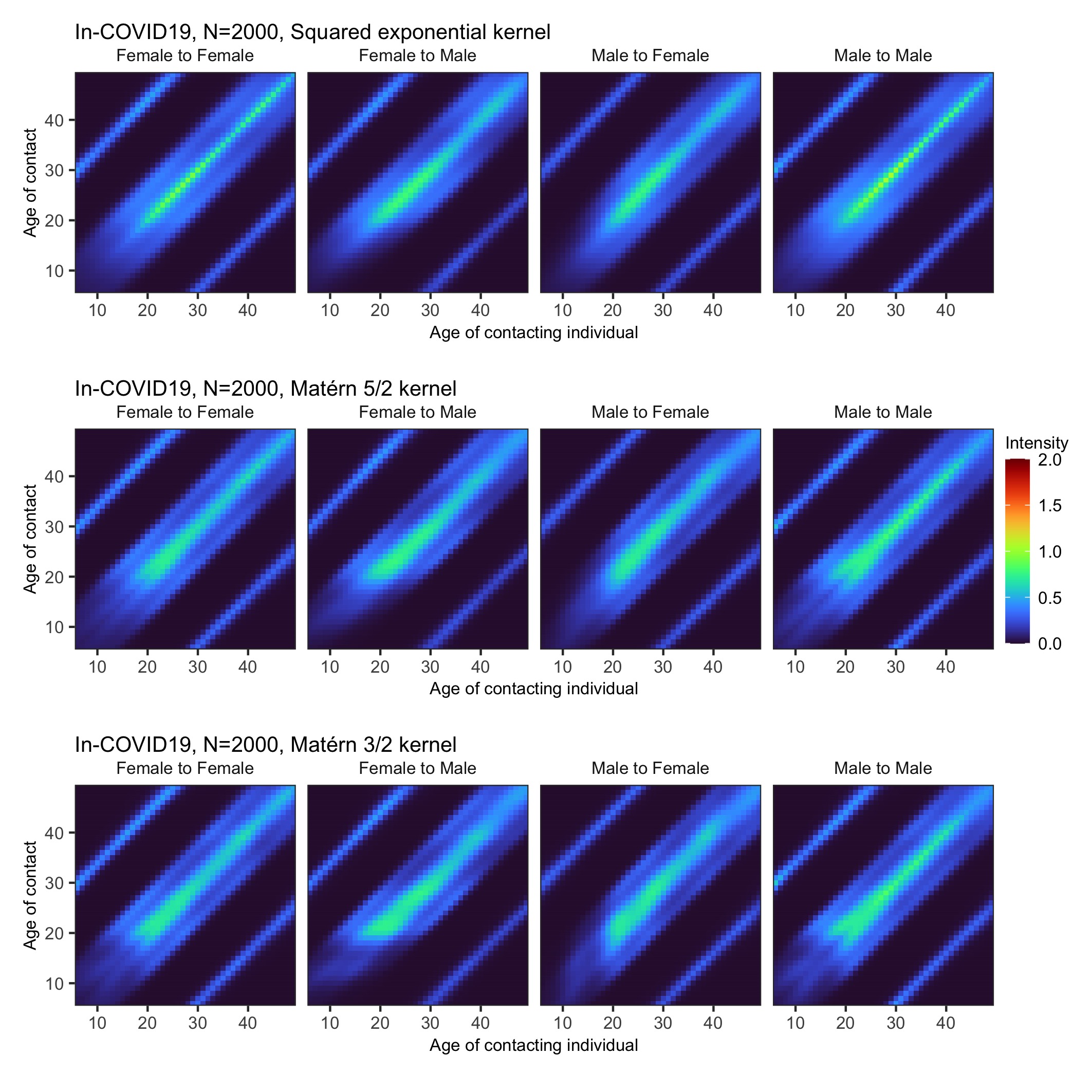

Supplement: S5 Fig — From top to bottom: results for the squared exponential kernel, results for the Matérn 52 kernel, and results for the Matérn 32 kernel. All experiments were run with HSGP using the difference-in-age parameterisation models with M1 = 40 (Number of eigenfunctions on the difference-in-age dimension) and M2 = 20 (Number of eigenfunctions on the contacts’ age dimension). The sample size was fixed at N = 2000. (JPEG) [file pcbi.1011191.s005.jpeg]

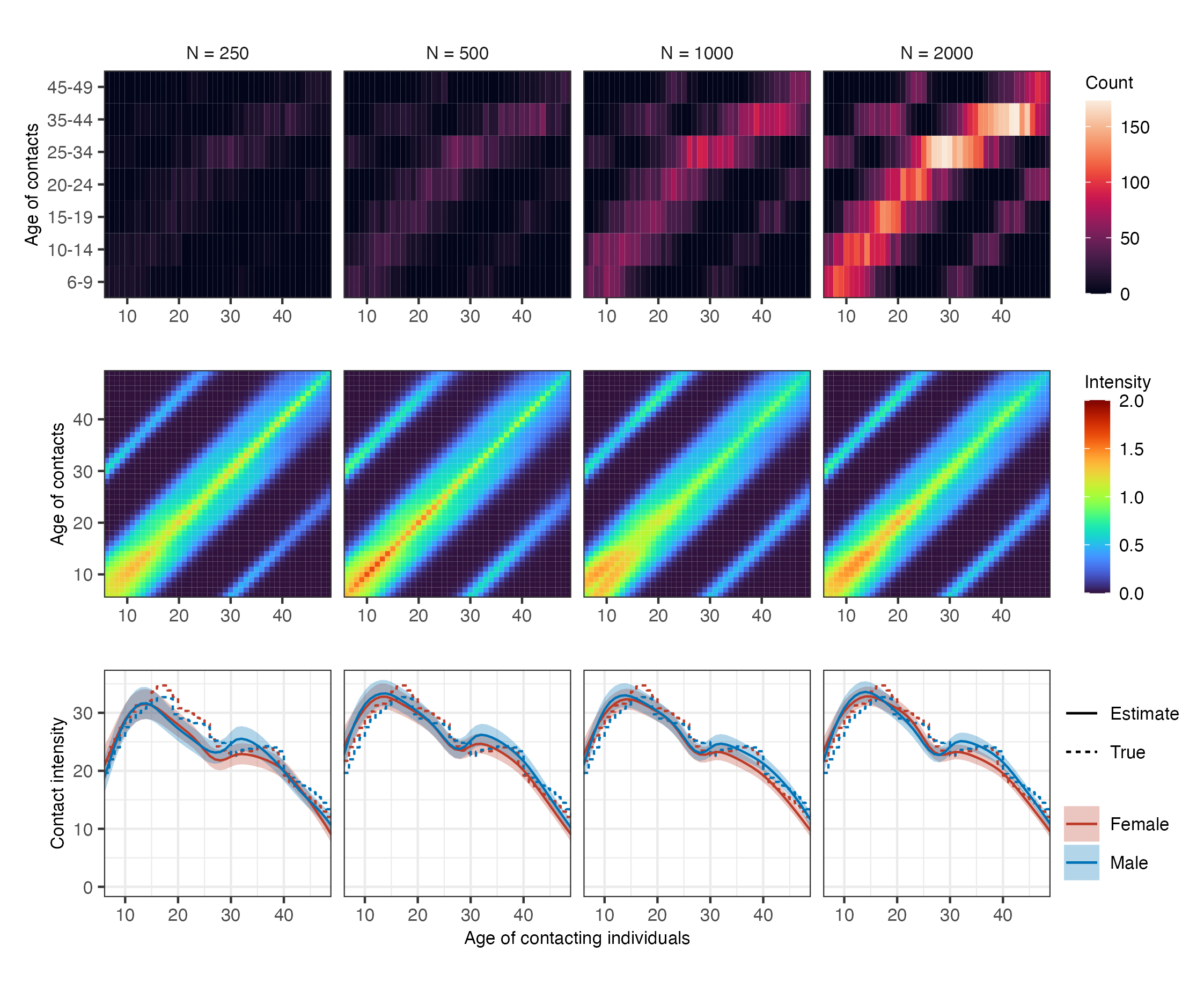

Supplement: S6 Fig — (Top row) Simulated contact data for the pre-COVID-19 scenario. (Middle row) Posterior median contact intensity estimates. (Bottom row) Posterior median marginal contact intensity estimates. Results were obtained using the Bayesian rate consistency model with difference-in-age parameterisation with M1 = 30 and M2 = 20. (PNG) [file pcbi.1011191.s006.png]

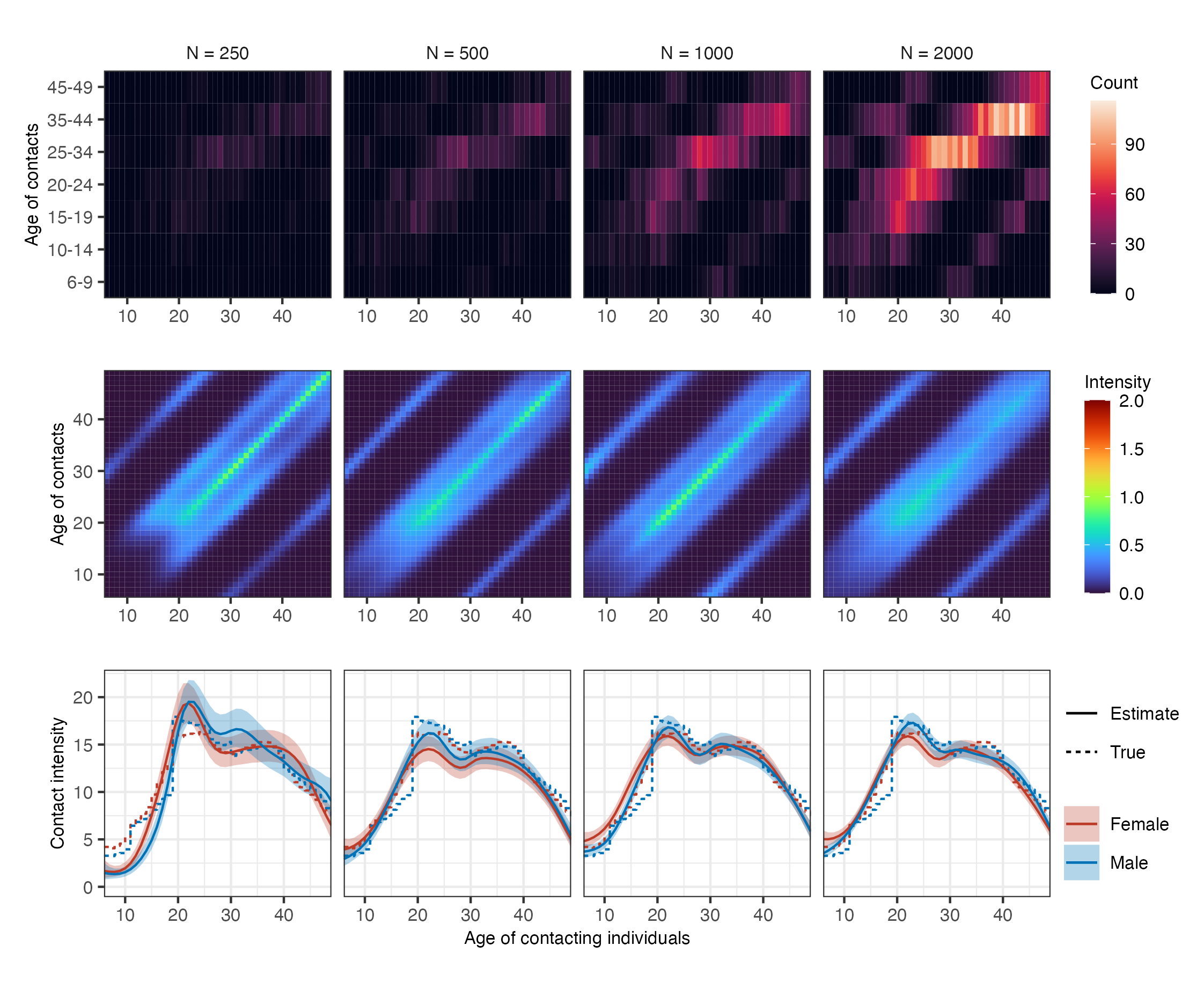

Supplement: S7 Fig — (Top row) Simulated contact data for the in-COVID-19 scenario. (Middle row) Posterior median contact intensity estimates. (Bottom row) Posterior median marginal contact intensity estimates. Results were obtained using the Bayesian rate consistency model with difference-in-age parameterisation with M1 = 30 and M2 = 20. (PNG) [file pcbi.1011191.s007.png]

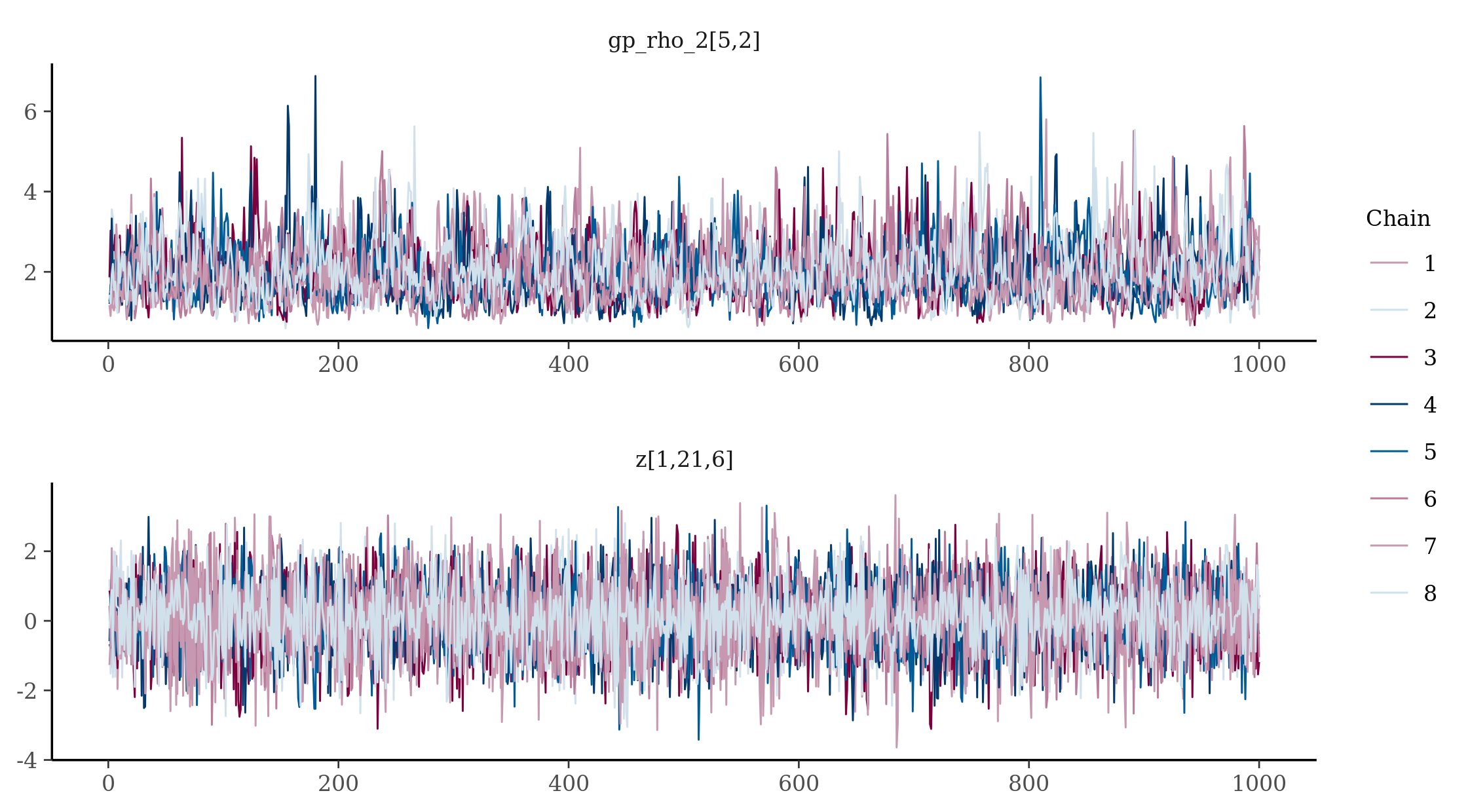

Supplement: S8 Fig — (Top row) Trace plot for parameter with the lowest effective sample size (1892), gp_rho_2[5, 2]: the length-scale parameter of the Gaussian process over the contact age dimension of the Female-Female contact matrix for wave 5. (Bottom row) Trace plot for the parameter with the largest R^ convergence diagnostic statistic (1.01), z[1, 21, 6]: the coefficient for the 21st HSGP basis function over the difference-in-age dimension of the Female-Female contact matrix for wave 1. (PNG) [file pcbi.1011191.s008.png]

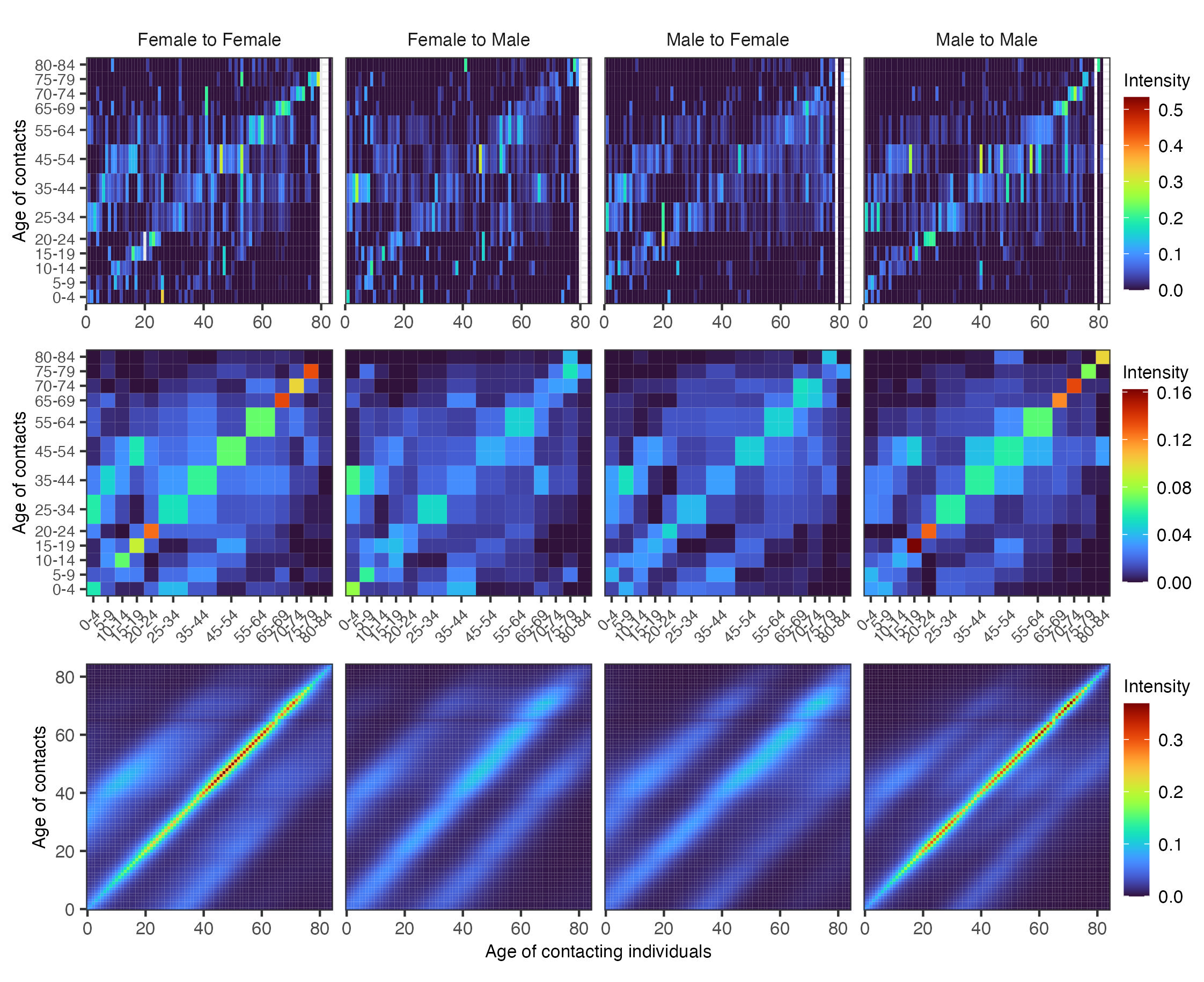

Supplement: S9 Fig — (Top row) Crude empirical social contact intensity patterns, with crude contact intensities above a value of 3 truncated for visualisation purposes. There are some age groups with no participants, and they are represented by white vertical columns. (Middle row) Contact intensity patterns as estimated by the socialmixr R package [11]. (Bottom row) Contact intensity patterns are given by our Bayesian model. (JPEG) [file pcbi.1011191.s009.jpeg]

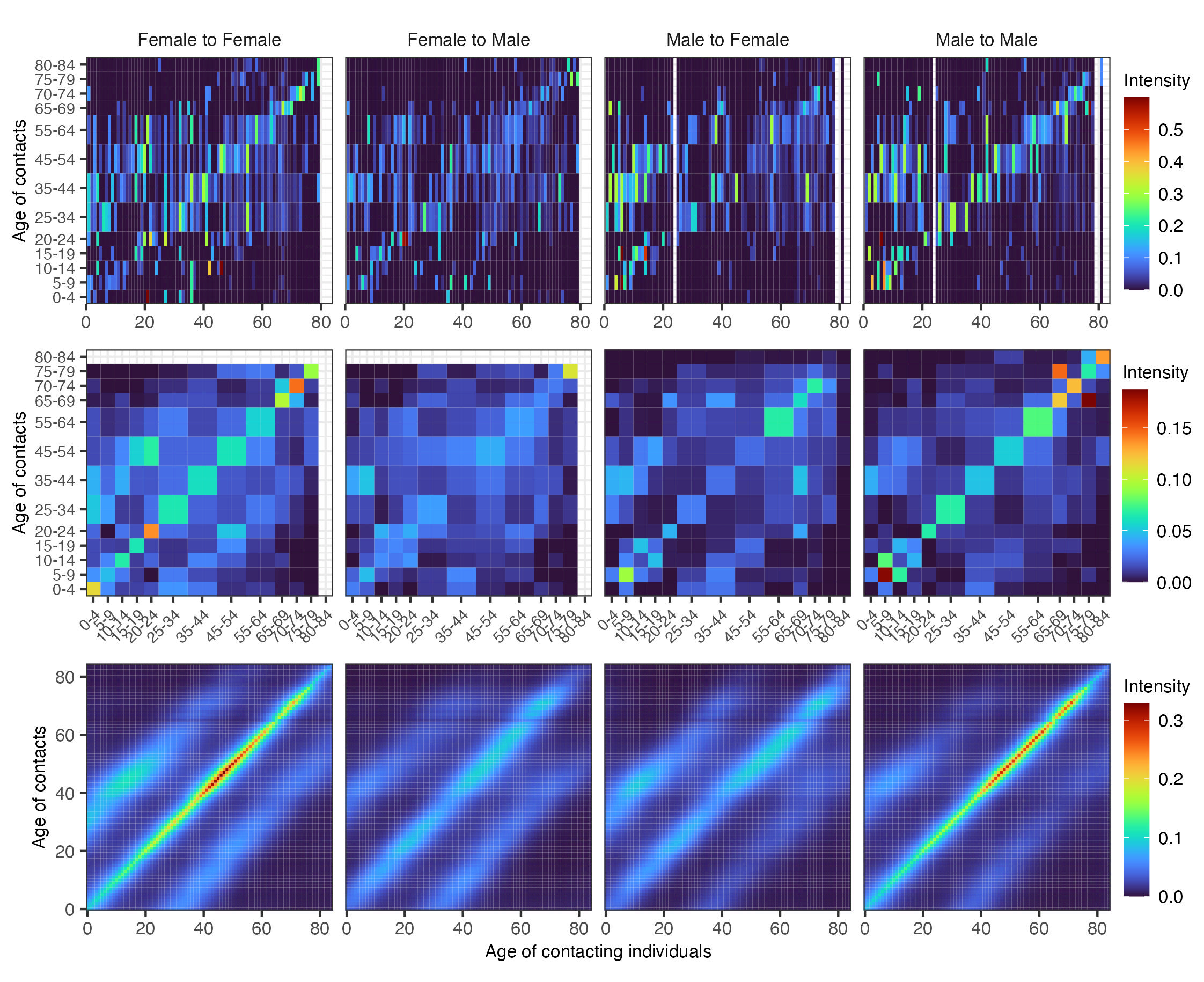

Supplement: S10 Fig — (Top row) Crude empirical social contact intensity patterns, with crude contact intensities above a value of 3 truncated for visualisation purposes. There are some age groups with no participants, and they are represented by white vertical columns. (Middle row) Contact intensity patterns as estimated by the socialmixr R package [11]. (Bottom row) Contact intensity patterns are given by our Bayesian model. (JPEG) [file pcbi.1011191.s010.jpeg]

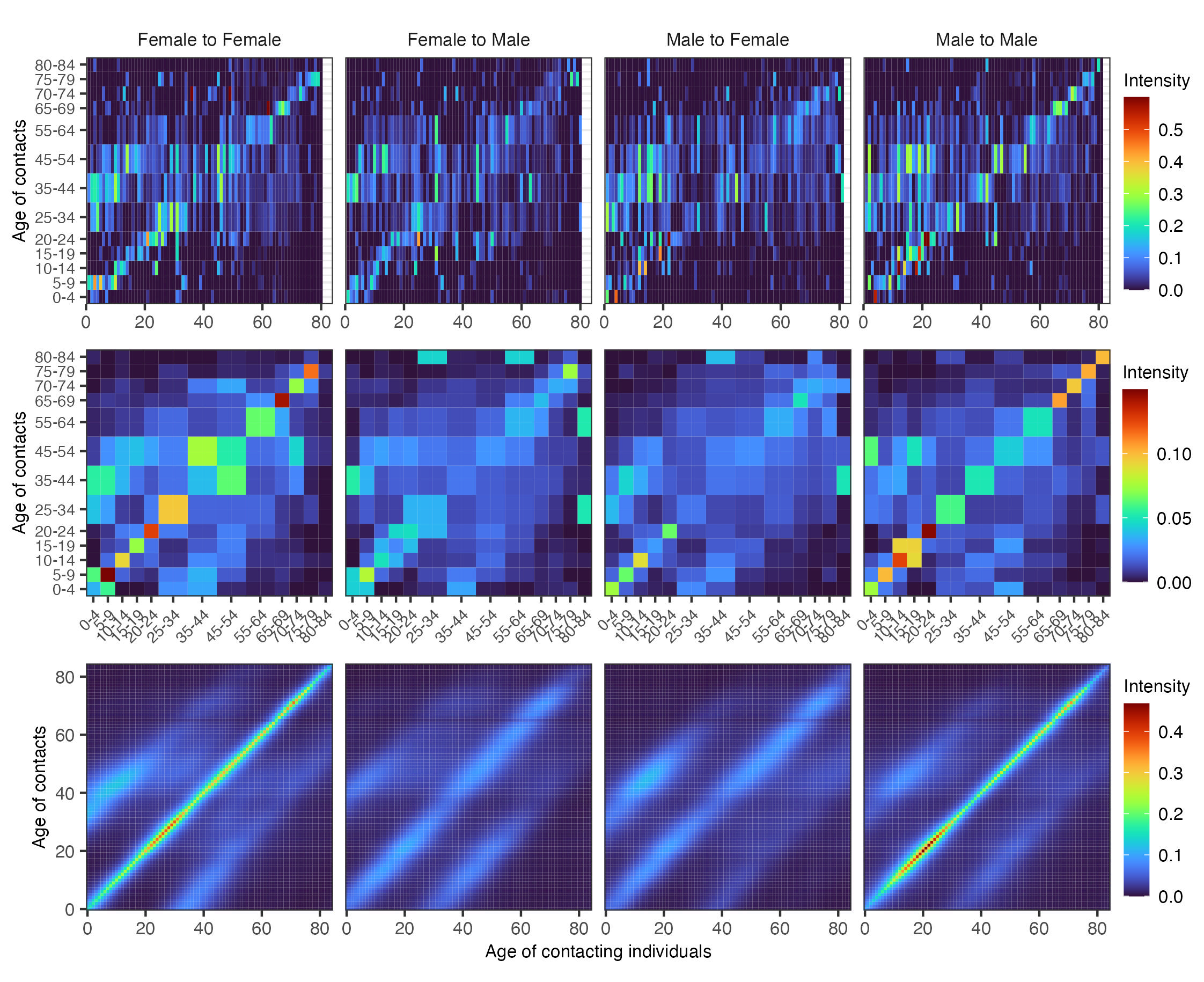

Supplement: S11 Fig — (Top row) Crude empirical social contact intensity patterns, with crude contact intensities above a value of 3 truncated for visualisation purposes. There are some age groups with no participants, and they are represented by white vertical columns. (Middle row) Contact intensity patterns as estimated by the socialmixr R package [11]. (Bottom row) Contact intensity patterns are given by our Bayesian model. (JPEG) [file pcbi.1011191.s011.jpeg]

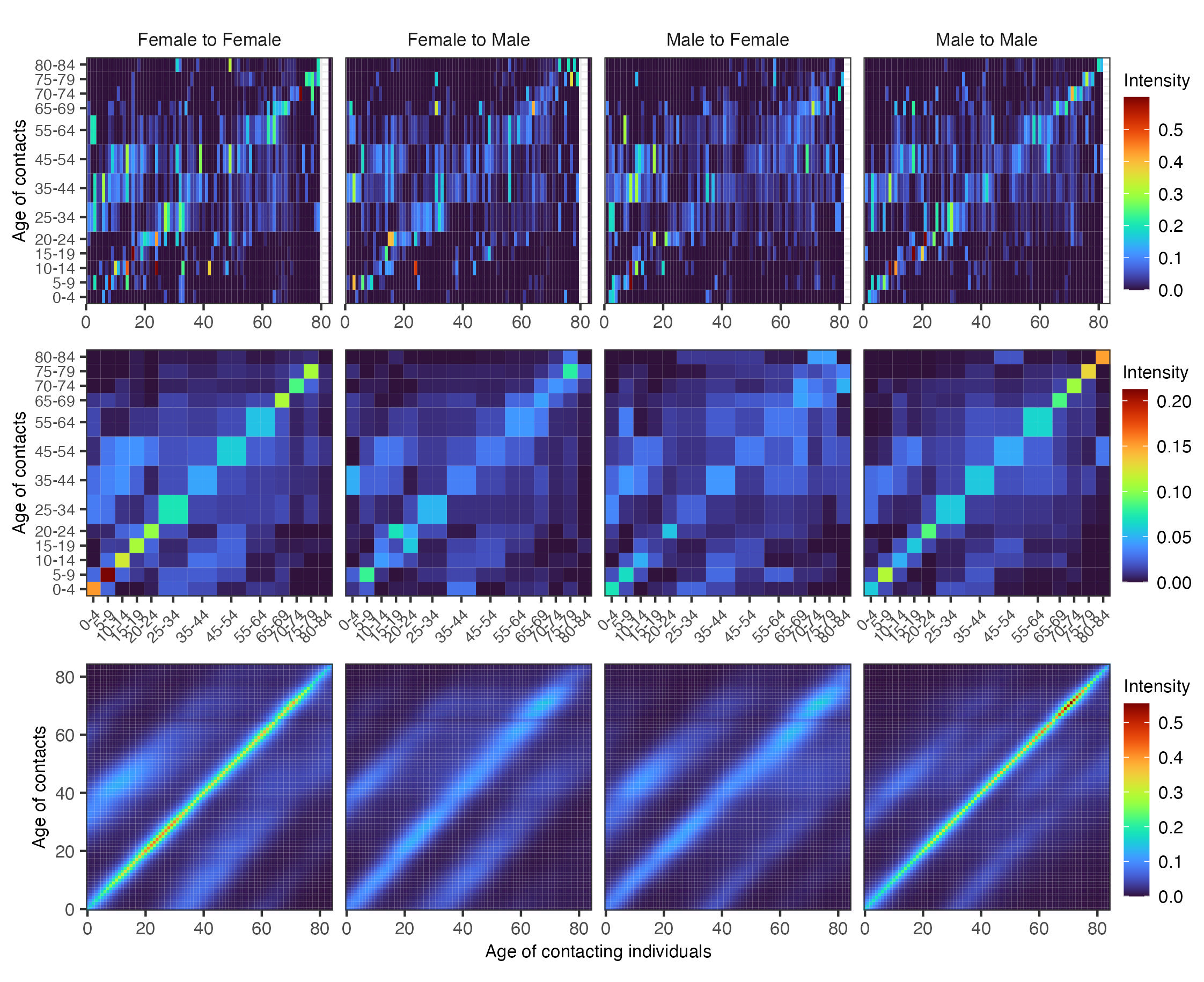

Supplement: S12 Fig — (Top row) Crude empirical social contact intensity patterns, with crude contact intensities above a value of 3 truncated for visualisation purposes. There are some age groups with no participants, and they are represented by white vertical columns. (Middle row) Contact intensity patterns as estimated by the socialmixr R package [11]. (Bottom row) Contact intensity patterns are given by our Bayesian model. (JPEG) [file pcbi.1011191.s012.jpeg]

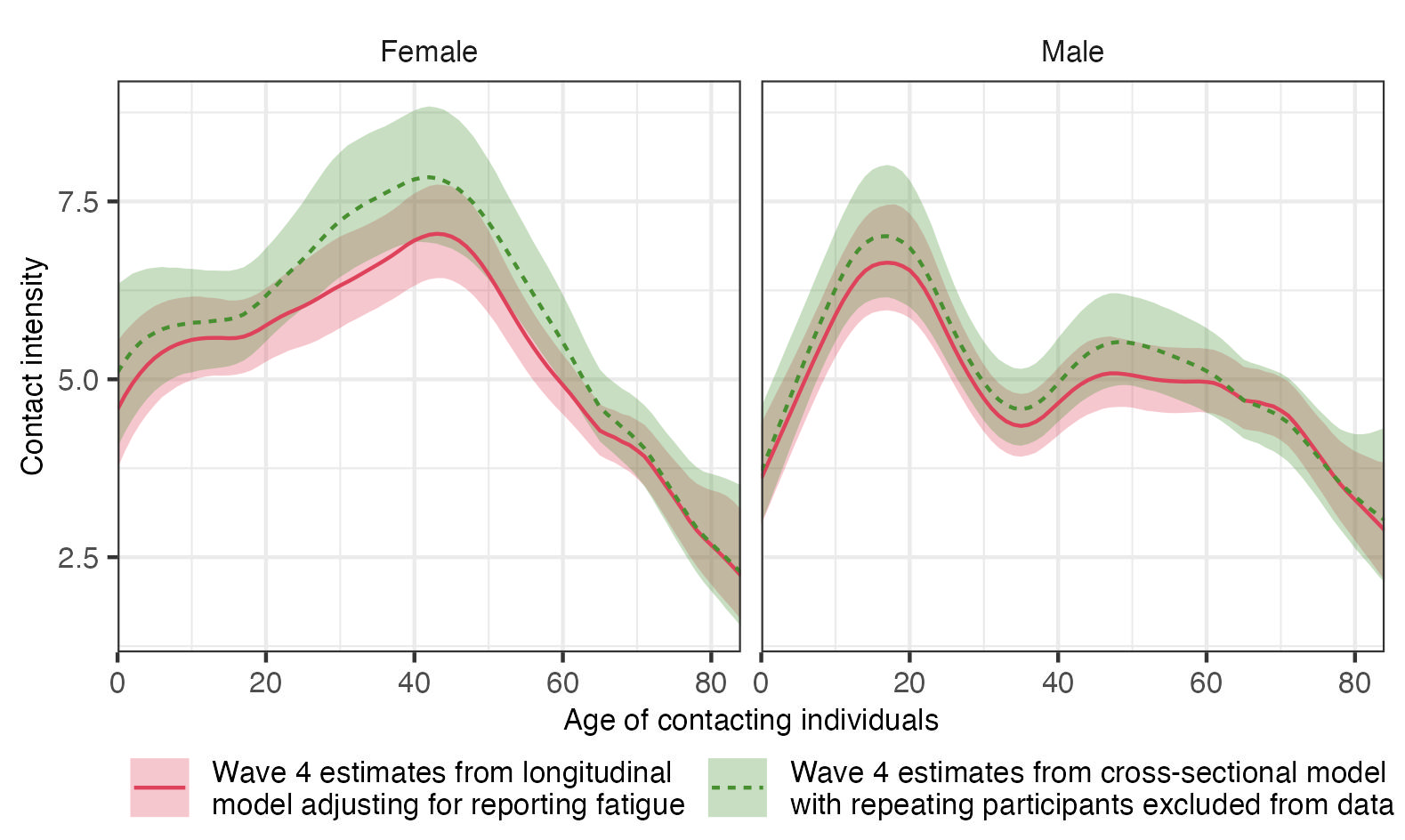

Supplement: S13 Fig — The red lines and ribbons show the posterior median estimates and 95% credible interval from the final longitudinal model on the entire COVIMOD dataset, adjusting for reporting fatigue. The green lines and ribbons display the estimated median and 95% credible interval from a cross-sectional model fitted on COVIMOD wave 4 data, excluding participants who answered the survey in previous waves. (JPEG) [file pcbi.1011191.s013.jpeg]

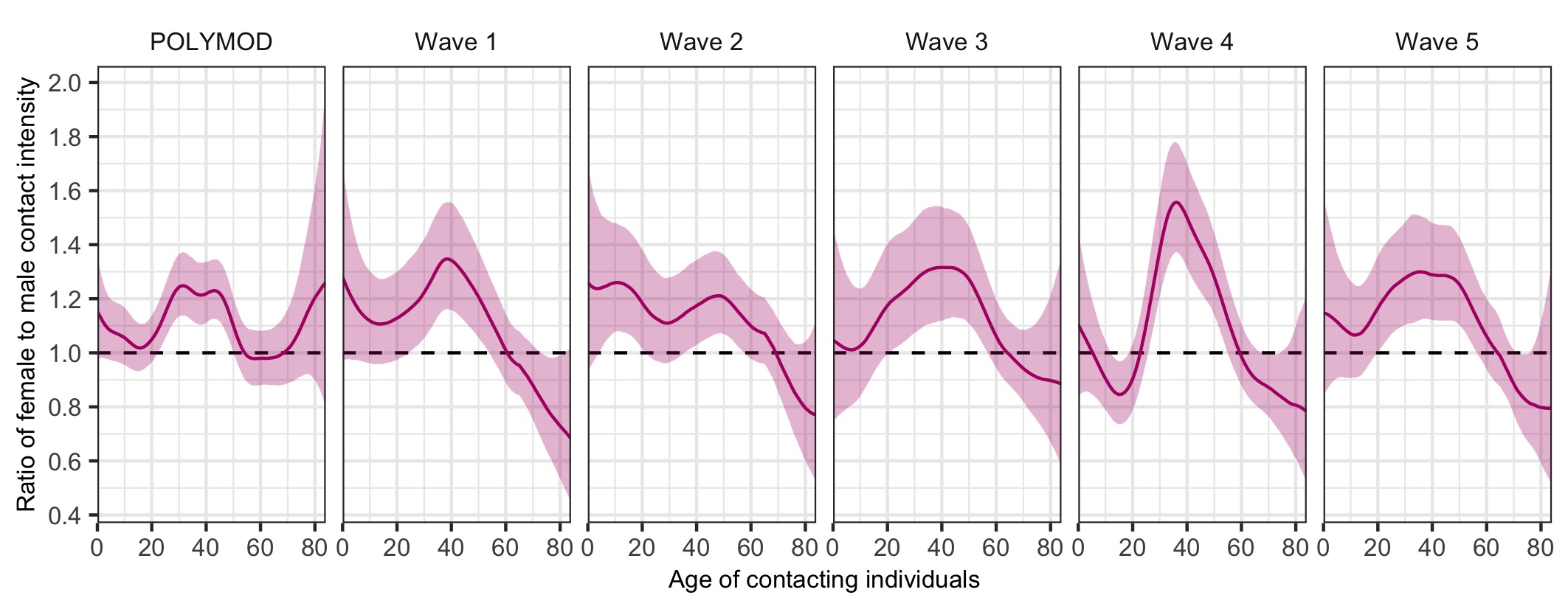

Supplement: S14 Fig — Lines represent posterior median estimates of the female-to-male marginal contact intensity ratios, i.e., maF/maM. A ratio of 1 (dashed lines) indicates no difference in contact intensities between genders. Shaded ribbons represent 95% credible intervals. (JPEG) [file pcbi.1011191.s014.jpeg]
