# Supplementary material for: Estimating fine age structure and time trends in human contact patterns from coarse contact data: The Bayesian rate consistency model
Source: PLoS Comput Biol. 2023 Jun 5;19(6):e1011191. doi: 10.1371/journal.pcbi.1011191 (PMC10270591; doi:10.1371/journal.pcbi.1011191)
Supplement: S1 Text — A detailed description of how the contact intensity patterns used in the simulation experiments are generated. (PDF) [file pcbi.1011191.s015.pdf]

S1 Text

Shozen Dan[1†◑*], Yu Chen[1†◑], Yining Chen[1], Oliver Ratmann[1◑*]

1 Department of Mathematics, Imperial College London, England, United Kingdom

◑These authors contributed equally to this work.
¤Current Address: Imperial College London, Exhibition Road, London SW7 2AZ, United Kingdom
* shozen.dan21@imperial.ac.uk, oliver.ratmann@imperial.ac.uk

The construction of simulated social contact patterns

Existing social contact studies showed that human social contacts are highly dependent on age and age-difference [1–5]. Specifically, they reveal that contact intensities are strong between people of the same age and between parents and children. We aimed to mimic these real-world social contact dynamics in creating our simulation datasets. For both the pre-COVID-19 and in-COVID-19 scenarios, we limited the age range to 6 to 49 for simplicity and to reduce the run time of our experiments. We designate individuals in age bands 6-18, 19-29, 30-39 and 40-49 as children, young adults, adults, and middle-aged adults, respectively. A subset of people aged 30-39 and 40-49 was assumed to be the parents of children and young adults. The following text describes the exact process of generating the simulated contact intensities. The R code may be found in script `R/sim-intensity-utility.R`, and the resulting contact intensity matrices can be found under `data/simulations/intensity` in the accompanying GitHub repository.

Pre-COVID-19 scenario

Based on findings from POLYMOD study [1], we set the number of social contacts per day in children to roughly 32, which includes the 2 contacts with their parents. We assumed young adults have approximately 25 contacts per day with peers (i.e., people in the same age category) and 2 contacts with their parents. Adults were assumed to have approximately 20 daily contacts with peers and 4 with children. Middle-aged adults were assumed to have approximately 15 contacts per day with peers and 4 with young adults. We set contact intensities to be the highest for age-assortative contacts (i.e., between the same age groups) and decreased the intensity as the absolute age difference (AAD) increased. Additionally, we increased contact intensity for parent-child contacts (the AAD is 24) and decreased the intensity as the ADD increased. The following text describes the full data generation set-up for the pre-COVID-19 scenario. The contact intensity of age pairs not included in the list was set to 0.

1. Participants aged 6-18:
   set the highest intensity as 2.5 ADD is zero, and decrease the intensity by 0.2 as the AAD increases to 8;
   set the intensity as 0.1 when AAD $\in [9:11]$;
   set the intensity as 0.03 when the AAD $\in [12:13]$;
   set the intensity as 0.01 when the AAD $\in [14:15]$;
   set the second highest intensity as 0.8 for AAD is 24, and decrease the intensity by 0.5 as the AAD-24 increases to 1;

set the intensity as 0.1 when the ADD-24 ∈ [2 : 3];
set the intensity as 0.01 when the AAD-24 ∈ [4 : 5].

2. Participants aged 19-29:
the highest intensity is 2.5 with 0.3 decrease by AAD until AAD = 5;
set the intensity as 0.8 when AAD ∈ [6 : 9];
set the intensity as 0.04 when the AAD ∈ [10 : 13];
set the intensity as 0.01 when the AAD ∈ [14 : 15];
set the second highest intensity as 0.8 for AAD is 24, and decrease the intensity by 0.5 as the AAD-24 increases to 1;
set the intensity as 0.1 when the AAD-24 ∈ [2 : 3];
set the intensity as 0.01 when the AAD-24 ∈ [4 : 5].

3. Participants aged 25-29, to generate the contacts with their children:
set the intensity as 0.2 when the AAD-24 is from ∈ [2 : 3];
set the intensity as 0.02 when the AAD-24 is from ∈ [4 : 5];
set the intensity as 0.6 when the AAD-24 is 1 and participants aged 29.

4. Participants aged 30-39:
the highest intensity is 2 with 0.24 decrease by AAD until AAD = 5;
set the intensity as 0.64 when AAD ∈ [6 : 9];
set the intensity as 0.03 when the AAD ∈ [10 : 13];
set the intensity as 0.01 when the AAD ∈ [14 : 15];
set the second highest intensity as 1.6 for AAD is 24, and decrease the intensity by 1 as the AAD-24 increases to 1;
set the intensity as 0.2 when the AAD-24 ∈ [2 : 3];
set the intensity as 0.02 when the AAD-24 ∈ [4 : 5].

5. Participants aged 40-49:
the highest intensity is 1.5 with 0.18 decrease by AAD until AAD = 5;
set the intensity as 0.5 when AAD ∈ [6 : 9];
set the intensity as 0.02 when the AAD ∈ [10 : 13];
set the intensity as 0.007 when the AAD ∈ [14 : 15];
set the second highest intensity as 1.6 for AAD is 24, and decrease the intensity by 1 as the AAD-24 increases to 1;
set the intensity as 0.2 when the AAD-24 ∈ [2 : 3];
set the intensity as 0.02 when the AAD-24 ∈ [4 : 5].

In-COVID-19 scenario

Using the pre-COVID-19 scenario as a baseline, we incorporated the effects of contact reduction measures such as school closures and remote work for the in-COVID-19 scenario based on findings from the CoMix study [4]. In children, the number of social contacts per day was set to roughly 3, among which 2 are with their parents. Young adults were assumed to have around 15 contacts per day with people of the same age and 2 with their parents. We assumed that adults have about 12 contacts with people of the same age and 2 with their children. Middle-aged adults were assumed to have approximately 10 contacts per day with peers and 2 with their children (young adults). Following the pre-COVID-19 scenario, contact intensities were set to be the highest for age-assortative contacts and decreased as the absolute age difference increased. Additionally, we increased contact intensity for parent-child contacts (the AAD is 24) and reduced the intensity as the ADD increased. The following text describes the full data generation setup for the in-COVID-19 scenario. The contact intensity of age pairs not included in the list was set to 0.

1. Participants aged 6-10:
   set the highest intensity as 0.08 ADD is zero, and decrease the intensity by 0.007 as the AAD increases to 8;
   set the intensity as 0.003 when AAD $\in [9:11]$;
   set the intensity as 0.001 when the AAD is $\in [12:13]$;
   set the intensity as 3×10^{-4} when the AAD $\in [14:15]$;
   set the second highest intensity as 0.8 for AAD is 24, and decrease the intensity by 0.5 as the AAD-24 increases to 1;
   set the intensity as 0.1 when the ADD-24 is $\in [2:3]$;
   set the intensity as 0.01 when the AAD-24 is $\in [4:5]$.

2. Participants aged 11-18:
   set the highest intensity as 0.4 ADD is zero, and decrease the intensity by 0.035 as the AAD increases to 8;
   set the intensity as 0.015 when AAD $\in [9:11]$;
   set the intensity as 0.005 when the AAD $\in [12:13]$;
   set the intensity as 15×10^{-4} when the AAD $\in [14:15]$;
   set the second highest intensity as 0.8 for AAD is 24, and decrease the intensity by 0.5 as the AAD-24 increases to 1;
   set the intensity as 0.1 when the ADD-24 $\in [2:3]$;
   set the intensity as 0.01 when the AAD-24 $\in [4:5]$.

3. Participants aged 19-29:
   the highest intensity is 1.5 with 0.18 decrease by AAD until AAD = 5;
   set the intensity as 0.48 when AAD $\in [6:9]$;
   set the intensity as 0.024 when the AAD $\in [10:13]$;
   set the intensity as 0.006 when the AAD $\in [14:15]$;
   set the second highest intensity as 0.8 for AAD is 24, and decrease the intensity by 0.5 as the AAD-24 increases to 1;
   set the intensity as 0.1 when the AAD-24 $\in [2:3]$;
   set the intensity as 0.01 when the AAD-24 $\in [4:5]$.

4. Participants aged 25-29, to generate the contacts with their children:
   set the intensity as 0.1 when the AAD-24 is from $\in [2:3]$;
   set the intensity as 0.01 when the AAD-24 is from $\in [4:5]$;
   set the intensity as 0.6 when the AAD-24 is 1 and participants aged 29.

5. Participants aged 30-39:
   the highest intensity is 1.25 with 0.15 decrease by AAD until AAD = 5;
   set the intensity as 0.4 when AAD $\in [6:9]$;
   set the intensity as 0.01875 when the AAD is $\in [10:13]$;
   set the intensity as 0.00625 when the AAD $\in [14:15]$;
   set the second highest intensity as 0.8 for AAD is 24, and decrease the intensity by 0.5 as the AAD-24 increases to 1;
   set the intensity as 0.1 when the AAD-24 $\in [2:3]$;
   set the intensity as 0.01 when the AAD-24 $\in [4:5]$.

6. Participants aged 40-49:
   the highest intensity is 1 with 0.12 decrease by AAD until AAD = 5;
   set the intensity as 0.33 when AAD $\in [6:9]$;
   set the intensity as 0.01375 when the AAD $\in [10:13]$;
   set the intensity as 0.00475 when the AAD $\in [14:15]$;
   set the second highest intensity as 0.8 for AAD is 24, and decrease the intensity by 0.5 as the AAD-24 increases to 1;

set the intensity as 0.1 when the AAD-24 $\in [2:3]$;
set the intensity as 0.01 when the AAD-24 $\in [4:5]$.

References

1. Mossong J, Hens N, Jit M, Beutels P, Auranen K, Mikolajczyk R, et al. Social Contacts and Mixing Patterns Relevant to the Spread of Infectious Diseases. PLOS Medicine. 2008;5(3):e74. doi:10.1371/journal.pmed.0050074.

2. Feehan DM, Mahmud AS. Quantifying population contact patterns in the United States during the COVID-19 pandemic. Nature Communications. 2021;12(1):893. doi:10.1038/s41467-021-20990-2.

3. van de Kassteele J, van Eijkeren J, Wallinga J. Efficient estimation of age-specific social contact rates between men and women. The Annals of Applied Statistics. 2017;11(1):320–339. doi:10.1214/16-AOAS1006.

4. Jarvis CI, Van Zandvoort K, Gimma A, Prem K, Auzenbergs M, O'Reilly K, et al. Quantifying the impact of physical distance measures on the transmission of COVID-19 in the UK. BMC Medicine. 2020;18(1):124. doi:10.1186/s12916-020-01597-8.

5. Monod M, Blenkinsop A, Xi X, Hebert D, Bershan S, Tietze S, et al. Age groups that sustain resurging COVID-19 epidemics in the United States. Science. 2021;371(6536):eabe8372. doi:10.1126/science.abe8372.
