# Supplementary material for: Estimating fine age structure and time trends in human contact patterns from coarse contact data: The Bayesian rate consistency model
Source: PLoS Comput Biol. 2023 Jun 5;19(6):e1011191. doi: 10.1371/journal.pcbi.1011191 (PMC10270591; doi:10.1371/journal.pcbi.1011191)
Supplement: S1 Table — Results were obtained with models using the difference-in-age parameterisation. The sample size was fixed at N = 2000 throughout. M1: The number of HSGP basis functions on the difference-in-age dimension. M2: The number of HSGP basis functions on the contacts’ age dimension. aMean absolute error, bExpected log posterior density, cPosterior predictive check, dMedian runtime, epre-COVID19, fin-COVID19 scenario. (PDF) [file pcbi.1011191.s016.pdf]

S1 Table. Comparison of performance on simulated data for different scenarios, covariance kernels, and the number of basis functions.

**Scenario**	**Kernel**	M^1	M^2	**MAE**^{a}	**ELPD**^{b}	**PPC**^{c}	**Runtime**^{d}
pre^e	SE	20	20	5.62×10^{-2}	-3120.4	98.4%	1.6 hours
pre	SE	30	20	5.00×10^{-2}	-3065.7	98.3%	1.1 hours
pre	SE	40	20	4.97×10^{-2}	-3064.2	98.3%	1.2 hours
pre	SE	40	30	4.97×10^{-2}	-3064.4	98.3%	1.6 hours
pre	Matérn 5/2	20	20	5.38×10^{-2}	-3099.4	98.6%	1.4 hours
pre	Matérn 5/2	30	20	4.79×10^{-2}	-3040.5	98.5%	2.0 hours
pre	Matérn 5/2	40	20	4.44×10^{-2}	-3027.6	98.5%	2.1 hours
pre	Matérn 5/2	40	30	4.41×10^{-2}	-3027.9	98.5%	3.1 hours
pre	Matérn 3/2	20	20	5.63×10^{-2}	-3094.6	99.0%	2.1 hours
pre	Matérn 3/2	30	20	4.81×10^{-2}	-3037.4	98.8%	1.4 hours
pre	Matérn 3/2	40	20	4.37×10^{-2}	-3024.2	98.9%	1.2 hours
pre	Matérn 3/2	40	30	4.36×10^{-2}	-3023.7	98.9%	1.5 hours
in^f	SE	20	20	3.73×10^{-2}	-2723.2	98.3%	2.3 hours
in	SE	30	20	3.27×10^{-2}	-2691.4	98.4%	1.1 hours
in	SE	40	20	3.27×10^{-2}	-2691.3	98.4%	0.7 hours
in	SE	40	30	3.27×10^{-2}	-2691.3	98.4%	0.9 hours
in	Matérn 5/2	20	20	3.56×10^{-2}	-2675.7	98.6%	1.3 hours
in	Matérn 5/2	30	20	2.97×10^{-2}	-2639.4	98.6%	1.1 hours
in	Matérn 5/2	40	20	2.85×10^{-2}	-2674.5	98.7%	1.4 hours
in	Matérn 5/2	40	30	2.85×10^{-2}	-2635.2	98.7%	1.8 hours
in	Matérn 3/2	20	20	3.41×10^{-2}	-2652.0	98.9%	2.7 hours
in	Matérn 3/2	30	20	2.86×10^{-2}	-2619.5	99.0%	1.2 hours
in	Matérn 3/2	40	20	2.70×10^{-2}	-2614.1	99.0%	1.4 hours
in	Matérn 3/2	40	30	2.69×10^{-2}	-2612.6	99.0%	1.6 hours

Results were obtained with models using the difference-in-age parameterisation. The sample size was fixed at $N = 2000$ throughout. M^1: The number of HSGP basis functions on the difference-in-age dimension. M^2: The number of HSGP basis functions on the contacts' age dimension. ^{a}Mean absolute error, ^{b}Expected log posterior density, ^{c}Posterior predictive check, ^{d}Median runtime, ^{e}pre-COVID19, ^{f}in-COVID19 scenario.
