# Supplementary material for: Estimating fine age structure and time trends in human contact patterns from coarse contact data: The Bayesian rate consistency model
Source: PLoS Comput Biol. 2023 Jun 5;19(6):e1011191. doi: 10.1371/journal.pcbi.1011191 (PMC10270591; doi:10.1371/journal.pcbi.1011191)
Supplement: Attachment — Submitted filename: response_to_reviewers_r1.docx [file pcbi.1011191.s017.docx]

Dear PLOS Computational Biology editorial team,

Thank you for considering our manuscript, “Estimating fine age structure and time trends in human contact patterns from coarse contact data: the Bayesian rate consistency model”, for publication as an original article to PLOS Computational Biology. We appreciate the opportunity to revise our manuscript again and sincerely thank you and the reviewers for the additional thoughtful comments - we believe they clarified and improved our manuscript immensely. The corrections made to our manuscript are as follows:

## Reviewer #1

**General comments**

**1 Comment:** This manuscript is attractive and well-written but methodologically underdeveloped. The full-Bayesian model should be expanded to account for temporal trends. Currently, f_t, tau_t, alpha_t, and l_t are treated as a priori independent, but the use of splines or GPs or linear models would help borrow information across survey windows and explicitly account for temporal trends. The same goes for the time-varying reporting effects.

**1 Response:** We thank the reviewer for this positive assessment and their time evaluating our manuscript. We believe the proposed model already presents a substantial methodological advance in that it allows inference of contact patterns by 1-year age bands from coarsely reported age of contacts. The methods section already introduces many model components, most notably the entire Bayesian model, aggregation to reported age bands of contacts, Kronecker decomposed HSGP approximations, and difference in age parameterisation. We agree that incorporating regularising modelling assumptions over time could render the model even more efficient, and we have added to the Discussion section how longitudinal analyses could be made more computationally efficient:

“Our work is not without limitations. … additional research is needed to scale up the approach to survey data from 30 waves or more. Potential avenues include HSGP modelling over the time domain within our approach, Integrated Nested Laplace Approximations, penalised spline-based regressions or even variational autoencoders.”

**Major comments**

**2 Comment:** Regarding the second sentence of the section "Data Processing", the method of imputing children's ages seems more like adding noise than actual imputation (which usually uses ancillary data to predict missing data). Since you're already working within a Bayesian framework, wouldn't it make more sense to infer these ages as latent variables? Otherwise, it is hard to understand the benefit of adding noise.

**2 Response:** We thank the reviewer for this comment. The exact age of participating children was indeed unavailable, and we imputed the exact age of participants aged 0-17. This is necessary because our model requires exact age inputs by 1-year age bands from participants to reconstruct contact intensities by 1-year age bands under the rate consistency constraints.

We agree that our imputation approach (draw 1-year age bands for each participant aged 0-17 at random from within the 5 to 10-year age bands) is adding noise. However, when we re-ran our model several times with different seeds, the results were indistinguishable from each other, as illustrated below. We, therefore, did not pursue further refinements on this point.

**3 Comment:** The choice of directly including (8) in (9) seems arbitrary. Wouldn't it be reasonable to include any function that favours Y and penalizes T?

**3 Response**: Thank you for this good comment. We have modified the text around Eq. (8) to clarify that this is the correct adjustment to make under the assumption of missing conditionally at random age contacts:

“Thus, we can calculate the proportion of contacts that are reported with detailed age information

S_{ta}^g = Y_{ta}^g / (Y_{ta}^g + T_{ta}^g)

and, assuming that the age of contacts is missing at random within each participant group of gender g, age a and wave t, use it as an additional offset term in the linear predictor”

**4 Comment:** I find the HSGP terminology a little bit troubling. Isn't it more accurate to call this a truncated Karhunen-Loeve expansion? Is HSGP a rebranding or is it really different?

**4 Response**: Thank you, and we are very happy to clarify this point. The construction of Hilbert Space approximate Gaussian Processes (HSGPs), proposed by Solin and Särkkä in 2020, is based on iterative applications of Mercer’s expansion to arbitrarily large powers of particular covariance operators (the negative Laplacian operator) and careful convergence arguments. This is why the HSGP approximation does not involve a series expansion of eigenvalues times eigenvectors that satisfy some integral equation, but instead, a series expansion of the spectral density evaluated at eigenvalues times specific eigenvectors that derive from particular Laplacian eigenvalue problems on a compact domain (Solin and Särkkä, 2020). Karhunen-Loeve expansions are a different class of GP approximations that in general, require one to solve numerically certain integral equations over the compact domain for each new value of kernel hyperparameters. One challenge is to ensure that the numerical solutions to these integral equations are stable when the covariance kernel is not smooth as in the case of the Matern kernels that we use. We note the interesting recent work of Greengard and ONeil, <https://arxiv.org/pdf/2108.05924.pdf> and their Algorithm 3. Another challenge is that KL expansions are less straightforward to implement in Stan than HSGPs. We made no changes to the manuscript.

**5 Comment:** I don't think the stringency index is ever defined or its relevance is ever stated. It is cited, but I shouldn't have to look it up elsewhere.

**5 Response:** Thank you for pointing out that the text never defined the stringency index. We have now added the following explanation around line 100 of the main text:

“Fig 1A and B, we show the sampling periods with the number of daily COVID-19 cases, cumulative COVID-19-related deaths, and the OxCGRT Stringency Index, which is a measure that captures the degree of containment and closure policies in place for COVID-19 on a scale of 0% to 100% [19].”

**6 Comment:** The sentence beginning "The overdispersion parameter" is not a sentence.

**6 Response:** We thank the reviewer for this comment. We have fixed this grammatical error as follows around line 167:

“The overdispersion parameter .. is strictly greater than zero .., such that the variance of the contact counts .. is greater than the mean.”

**7 Comment:** What is the main point of the second paragraph within the "Difference in Age Parameterization" section? Is "this works better and is worth the additional computational cost"? Please clarify.

**7 Response:** Thank you for making this point. We have clarified the section and, in particular, the final sentence around line 272, as suggested:

“We will show in the results section that this parameterisation is crucial to obtaining accurate estimates of typical fine-age diagonal and off-diagonal human contact patterns from coarsely reported age data, and so recommend its use despite higher computational cost.”

## Reviewer #2

**General comments**

**8 Comment:** This paper introduces a novel method to analyse contact survey data where the age characteristic of the contact has only been reported as an age group. The method allows the estimation of contact matrices at a finer age scale. The resulting matrices remind me of approaches where a contact matrix of aggregate age groups has been smoothed (e.g. <https://doi.org/10.1186/1471-2334-9-187>), but the method presented here arguably makes better use of the precise recording of contactor ages. In addition, the authors aim to adjust their estimates by reporting fatigue, and for data collected at an aggregate level. This is then applied to analyse social contact data from Germany.

The authors used zero-mean two-dimensional Gaussian Processes to estimate the contact intensities between two ages. I am not familiar enough with this method to accurately review this technique. I have assumed that it is valid and appropriate in my review but would recommend a specialist review to look at this aspect of the method.

Overall, this is a very interesting paper and a useful addition to the literature on contact matrices. I do have a number of comments, mainly on i) the adjustment for the aggregated number of contacts, and ii) the simulations used to validate the model.

**8 Response:** We thank the reviewer for this positive assessment and are grateful for their time in evaluating our manuscript.

**Major Comments**

**9 Comment:** Both the incidence of cases and stringency index remained relatively stable between waves 2 to 5 (figure 1). However, your model estimates a gradual increase in contacts between these periods (figures 5, 6, and 8).

This is contradictory, as you would roughly expect changes in the force of infection and incidence to be proportional to the change in the number of cases.

**9 Response:** This is an excellent point which puzzled the scientific community involved in the global COVID-19 response in the summer 2020, including the Imperial College COVID-19 Response Team. Indeed, early predictions on the likely impact of relaxing pharmaceutical interventions were incorrect because gradual increases in mobility did not translate into proportional increases in the effective reproduction number and resulting exponential increases in deaths (IHME COVID-19 Forecasting Team, Modeling COVID-19 scenarios for the United States. *Nat. Med.* 27, 94–105 (2021); *P. Nouvellet et al., Report 26 - Reduction in mobility and COVID-19 transmission Imperial College London COVID-19 Reports* (2020); <https://doi.org/10.25561/79643>; A. C. Miller *et al.*, Mobility trends provide a leading indicator of changes in SARS- CoV-2 transmission. <https://doi.org/10.1101/2020.05.07.20094441>). Several explanations have been put forward, including behaviour change and mask use, changes in the structure of the age-specific contacts (as Rt is calculated from the dominant eigenvalue of the WAIFW matrix), or changes in the heterogeneity in the population-level contact distribution over time. To improve pandemic preparedness, it will be necessary to understand better the relationship between effective reproductive numbers and all these factors, for which we believe a better understanding of the patterns and trends in social contacts is essential.

**10 Comment:** In Figure S1, I would expect non-household contacts to increase as NPIs are relaxed. However, they remain stable, while mainly the aggregated contacts seem to increase over time.

Part of that is artificial because they could only be reported from wave 3 onwards (line 89 – please explain why there are also aggregate contacts in wave 2 in your figure S1).

But I would expect to see a similar increase in non-household contacts if there was indeed a true change in behaviour increase.

**10 Response:** We thank the reviewer for this comment and apologise for the confusion in Supplementary Figure S1. There were aggregate contacts in wave 2 because we show all individual contact reports with missing age and gender information and those reported in aggregate in red. The corresponding label in Supplementary Figure S1 and the legend text were incorrect, which we have now fixed.

Regarding the last point made, we note that in the Netherlands CoMix study (Backer et al.), increases in the number of contacts are evident from the second half of May onwards (their Figure 3). Coletti et al. observed similar patterns in Belgium, with increases in the number of contacts reported from the 3rd week of May onwards (their Figure 1). Considering our study period from May to July 2020, increases are less evident from Figure 1b-c in Gimma et al. for the UK. Figure 1 in Gimma et al. is scaled to accommodate the significant increases in mean contacts among school-aged children in September. Still, a closer look suggests an absolute increase of 1-1.5 contacts per day among participants aged 18-59 by the end of June. Such an increase is consistent with our estimates that account for missing & aggregate contacts but do not account for reporting fatigue (our Figure 5, wave 5, light blue dashed line). We hope this clarifies our analysis and we have further clarified our discussion around line 495:

“We found relatively limited rebounds in contact intensities after lifting contact reduction measures and that social contact intensities remained substantially below pre-pandemic values until the end of our study period in July 2020 (Fig 6). These results are consistent with social contact survey data in England [13], Belgium [14], the Netherlands [15], and elsewhere. […] Yet, over the first five COVIMOD survey waves between May and July 20202, we also observed structured changes in contact patterns, with social contact intensities rebounding more rapidly with the increasing age of individuals (Fig 7). These increases in social contacts were also far from uniform across population age bands of contacted individuals. Most increases tended to centre on the off-diagonals of the age-age social contact intensity matrix (Fig 8).”

We have also taken the opportunity to investigate trends in individually reported non-household contacts further, individually reported non-household contacts + missing & aggregate contacts, and all contacts reported. For this analysis, we considered only first-time participants to mitigate reporting fatigue effects in the empirical data. We considered data from wave 1 and wave 4 during which sufficiently many participants were first-timers. We also considered for comparison trends in the number of contacts in all participants, regardless of being first-timer or not. The left figure below shows clearly that there was a significant increase in the number of contacts from wave 1 (early May) to wave 2 (mid-June) in non-household contact, with missing & aggregate contacts excluded. The left figure also shows that the increase in the contact intensities that we report is predominantly driven by contacts reported in aggregate or with missing detail on the age of the contact. We added the following sentence around line 465:

“Fig 7 shows that the contact intensities increased consecutively from wave 1 to wave 5, with the increases being most substantial from wave 1 to wave 2. These increases are primarily due to marked increases in the number of contacts that were reported in aggregate over the first five survey waves (Supplementary Fig. 3).”

**11 Comment:** I have some doubts about the data quality and accuracy of the reported aggregate number of contacts. Direct contacts are relatively clearly defined (physical touch and/or a short in-person conversation), but people can also have other in-person contacts that do not necessarily fit this definition (e.g. shop clerks interacting with customers). These other contacts are not as clearly defined but are often referred to as indirect contacts. Many surveys, including POLYMOD, allowed participants to roughly estimate such indirect contacts. They are probably less important for transmission than direct contacts (no/fewer words spoken, usually of shorter duration or larger physical distance).

The aggregate contacts referred to in this paper may also be an example of such indirect contacts (I could not see the actual wording of the questions asked), and I think that this should be explored further:

At a minimum, could you report the proportion of aggregate to individually reported contacts in each age and wave?

**11 Response:** We thank the reviewer for this comment and suggestion. The aggregate contacts reported in the COVIMOD survey correspond to the indirect contacts reported in POLYMOD and those in the related CoMIX surveys. The English translation of the question asked within the COVIMOD survey is thus:

“How many people have you had contact with whom you did not specify individually? Please provide an estimated value as precisely as possible, including age and environment. For this question, we are only interested in people with whom there was direct contact and with whom you have exchanged at least a few words OR with whom you have had physical contact (e.g. shaking hands, hugging, contact sports). Please, indicate the number of contacts for each age group and each environment.”

For reference, the question in the POLYMOD survey was thus:

“We do ask you to include every contact you had, but if you were unable to include every single contact (for instance, because you work in a shop till and have a large number of contacts in a day), please could you indicate this?”

“If you did not include every person you had contact with, approximately how many people you left out did you have?”

We clarified the text describing these contacts around line 90 as follows:

“From wave 3 onward, participants were given the possibility to report a total number of contacts that were either physical or non-physical contacts in the sense described above, and which they did not report individually, which we refer to as aggregate contacts.”

We agree that the aggregate contacts could potentially be less relevant for the transmission of respiratory pathogens. However, the COVIMOD and CoMix survey questions emphasise our interest in the number of direct contacts. For this reason, we opted to include these reports in our analysis, similar to previous studies from the Netherlands, Belgium and the UK. We also agree it is important to be transparent about the data sources and have added the requested plot as Supplementary Figure S2.

**12 Comment:** In lines 93, 140, and 205: It is not clear how participants with aggregate contacts are treated. Are the contacts added in Y_ab? Or do only the participants add to N_a, but their aggregate contacts not to Y_ab? If a person aged reports 20 aggregate contacts, does that mean 20 is added to T_ta? Please clarify this in the text.

If they are added to T_ta (and thus given equal weight as contacts in Y_ab), it would be useful to explore treating them differently, either by excluding them from the analysis or by giving them a lower weight than the direct contacts (it can potentially be fitted as a parameter in your model, you will have some information if you assume people of similar age have on average the same number of daily contacts, and there are sufficient people with/without aggregate contacts).

**12 Response:** We thank the reviewer for raising this issue, which is important to clarify. We have modified the text around Equation 8 as follows:

“A second challenge is that the number of missing & aggregate contact reports also fluctuated over time. As denominators in our sample, we now consider all participants of age a and gender g regardless of whether they reported individual contacts or missing & aggregate contacts and denote their total in wave t with repeat participation r by N^g_{tra} as in (7c). We further denote the number of missing & aggregate contact reports by participants of age a and gender g in survey wave t by T^g_{ta}. For instance, if a participant in wave t of age a and gender g had two contacts with missing age or information and reported 18 aggregate contacts, we added 20 to T^g_{ta}.”

The counts Y_ab included only individual contacts for whom the age and gender were specified. We believe this part was already transparent in the text:

“In addition, consider the number of total contacts with detailed age information by participants of age a and gender g in survey wave t by Y_{ta}^g = \sum_{r,c, h} Y_{trac}^{gh}.”

and made no further clarifications on this part. Please let us know if any further clarifications would be desirable and we will be happy to make these. The result of excluding this offset is shown in figure 5, where we ran our model adjusting for reporting fatigue but not for missing & aggregate contacts.

**13 Comment:** The simulated datasets seem to generate realistic contact matrices with strong age-assortative mixing and off-diagonals representing child-parent contacts. The model seems to be able to estimate this matrix quite well, where the difference-in-age method is better than the age-age method. However, that is perhaps not surprising, as the data is also generated to make sure that there are strong difference-in-age patterns.

Real-world data is a lot messier, and it would be useful to see how well the model performs against a real-world dataset where the age of the contact is known. POLYMOD has the age in years (as an integer) for both participants and most contacts. It would be useful to understand how well the model can estimate the age-granular POLYMOD matrix (assuming that it is true) from POLYMOD data converted to data in age groups. In addition, mixing patterns can look very different in populations that do not follow a rectangular age distribution (such as Germany or the UK), but where the age distribution is pyramid-shaped as is the case in many countries with high mortality rates (e.g. Kenya or Malawi). In these populations, age-assortative mixing is often less apparent in matrices as contacts between children dominate the overall contacts. It would be very useful to understand how well the model behaves for matrices in these populations.

**13 Response:** We thank the reviewer for this great suggestion. We ran experiments where we aggregated age-granular POLYMOD data into age groups to understand how well the model can estimate the age-granular matrix. We have also run experiments using data from Manicaland, Zimbabwe, which has a pyramid-shaped population age distribution. We have expanded the first Results paragraph, “Contact patterns by 1-year age band can be estimated”, as follows:

“In several previous social contact surveys, participants were asked to report the exact age of their contacts. This allowed us to further test the Bayesian rate consistency model by artificially withholding the exact age of each contact in real-world data. Specifically, we tested how closely the reconstructed fine-age contact intensities inferred on the masked data with the age of contact masked into broad age bands match those inferred from the actual data with the exact age of contacts known. Figure~\ref{fig:polymod_exact_age} shows the outcomes of this test on data from 1,292 participants who reported 31,670 contacts in Germany as part of the POLYMOD study~\cite{mossong_social_2008}. The contact intensities learned from the data with the exact age of contact and those learned from the data with age of contact masked into broad age bands were qualitatively very similar, and the mean absolute difference across each age-age gender-gender group was 0.145 in the context of a mean contact intensity of 0.120 in each age-age gender-gender group. We repeated this test on data from 1,122 participants from the Manicaland HIV/STD Prevention Study~\cite{melegaro_social_2017}, a general population cohort study carried out since 1998 in Manicaland, the easternmost province of Zimbabwe. In 2013, participants were asked to report on their social contacts, which were either physical or non-physical, in two-way conversations with three or more words in the physical presence of another person, and the exact age was reported for 24,480 contacts. We chose these data for a second test because Manicaland, Zimbabwe's population structure and social contact patterns differ markedly from those seen in Europe~\cite{mossong_social_2008, melegaro_social_2017}. Figure~\ref{fig:manicaland_exact_age} shows our test outcomes on the Manicaland data. The Bayesian rate consistency model generated again very similar estimates on the data with the age of contact masked into broad age bands as compared to the actual data with the exact age of contact known, and the mean absolute difference across each age-age gender-gender group was 0.179 in the context of a mean contact intensity of 0.125 in each age-age gender-gender group.”

**Minor Comments**

**14 Comment:** 17: More difficult compared to what exactly?

**14 Response:** We thank the reviewer for this comment. We have rewritten the sentence as such:

“It is important to estimate high-resolution age- and gender-specific human contact patterns and their trends over time, but this has remained an unsolved challenge due to changes in survey methodology from pre-pandemic studies […] and limitations in available inference methods […].”

**15 Comment:** 22, 40: The authors write that participants record contact ages in large age categories in COVID-era studies to facilitate reporting. In many surveys, this was a restriction decided on by the survey implementor (the online market research platform) for ethical and privacy reasons. Note that it is not unique to COVID-era studies, e.g. POLYMOD also allowed participants to provide an age range if the exact age of contact was not known (which is common).

**15 Response:** We thank our reviewer for this helpful clarification. We have modified the text to avoid confusion as follows. Around line 22:

“First, COVIMOD and other COVID-era social contact studies record the age of contacts by large age bands of 5 to 10 years, reflecting that often it is difficult for study participants to know the exact age of their contacts. In contrast, the pre-pandemic POLYMOD surveys primarily collected data on the exact age of contacts.”

Around line 40:

“Additionally, participants [DELETED: “in COVID-era surveys”] sometimes found it difficult to recall specific age and gender information for all of their contacts. Instead, they were allowed to report an estimate for the total number of contacts on that occasion~\cite{tomori_individual_2021}, which again may result in under-ascertainment of contact intensities if these data are not accounted for in inference approaches of contact patterns.”

**16 Comment:** 120, 146: Why did you cut your matrices off above age 84? Are participants and contacts above these ages grouped together, and should the final age group be interpreted as a single 84+ group?

**16 Response:** Thank you for this question. We cut off the matrices at 84 because we deemed that there were not enough samples of participants above 84 to ensure accurate inference. The final age group should not be interpreted as an 84+ age group. We have clarified the main text around line 120 as follows:

“After excluding participants who prefer not to provide age or gender information and 25 participants above the age of 84,...”

**17 Comment:** Line 133: when imputing the age of the child, did you create multiple datasets to fit? Did you redraw the age in each MCMC iteration?

**17 Response:** Thank you, please note this question is similar to comment 2 from our other reviewer. We created multiple datasets to fit using different random seeds and inferred contact intensities separately for each data set. We found this had only a minute impact on our estimates, given the available sample sizes of participants aged 0-17 in the national survey and therefore did not pursue further any refinements on this point. In our response to comment 2 above, we provide more detail on the small impact of the different imputations on our estimates.

**18 Comment:** Line 138: Unclear what is meant by a missing entry. I assume it means that not a single participant of age a and gender g reports any contact with contacts of age b and gender h, but this is not clear.

**18 Response:** We thank the reviewer for pointing out this flaw in our writing. We have revised the relevant sentences as follows around line 90:

“For survey waves 1 and 2, participants were asked to provide each contact’s information separately. However, some participants reported contacts to groups of individuals (e.g., customers, clients) for which a specific number of contacts was assumed (Additional file 2 of [9]). From wave 3 onward, in addition to being asked to report contacts individually, participants were given the possibility to report a total number of contacts that were either physical or non-physical contacts in the sense described above and which they did not report individually, which we refer to as aggregate contacts. Additionally, some participants could not recall or preferred not to answer the age or gender information of some of the individual contacts that they reported. We treat these three types of entries with missing age or gender equally and refer to them as missing & aggregate contact reports.”

And around line 143:

“Finally, regarding the three types of missing & aggregate contacts reported, i.e. contacts reported to groups of individuals, additional contacts reported for who participants did not provide individual information, and individual contacts reported for who participants did not provide age and gender information, we truncated these at 60 (90th percentile) to remove the effects of extreme outliers. We show the distribution of observed contacts in S1 Fig and S2 Fig.”

**19 Comment:** 156, 161: be consistent when describing contact rates, e.g. make it clear that this is the rate per unit time (probably day)

**19 Response:** Thank you. We have added the phrase “within a 24-hour time window” to our contact intensity and contact rates descriptions to remove the ambiguity just above and below Equation (1).

**20 Comment:** Useful to double-check notations. Especially M, F and g, h subscripts are used interchangeably, which is not always necessary.

**20 Response:** Thank you for this comment too. We have double-checked all instances of M and F and use this notation only to make the explanations more readable and intuitive. We are happy to reconsider any instances that take work to follow.

**21 Comment:** 187, 191: It may be better to speak of survey wave rather than time or survey time

**21 Response:** We thank the reviewer for the great suggestion. We have updated the text in the “Estimating dynamics in contact patterns” section that starts around line 189.

**22 Comment:** 283: I can’t find any trace plots in the supplement. Not possible to assess the mixing and convergence of chains. Would be useful to have a table with all summary statistics of the chains as well (for the final model).

**22 Response:** We thank the reviewer for this suggestion. We now include as Supplementary Figure 11 the trace plot of the model parameter with the smallest effective sample size. We also state in the main text around line 287 that

“The smallest effective sample size was 1892, and the Rhat convergence diagnostics were below 1.01, indicating that the Markov chains converged and mixed well.”

**23 Comment:** 293: small typo: studies are duplicated

**23 Response:** We thank the reviewer for notifying us about this error. We have fixed the text accordingly.

**24 Comment:** Table 1: it is hard to understand how the accuracy of the method changes from the summary statistics alone. Could these matrices also be plotted in the supplement?

**24 Response:** We thank the reviewer for this suggestion. We have included the contact matrices and comparison between the true and estimated marginal contact intensities as new Supplementary Figures 7 and 8.

**25 Comment:** Line 368: did you use the same population distribution in adjusting the socialmixr estimates for symmetry as has been used in your model (your P_a parameter)? This will (slightly) affect the final estimated contact matrix.

**25 Response:** We thank the reviewer for this comment. We used the same population distribution in adjusting for symmetry in socialmixr.

**26 Comment:** Line 370: contacts with missing age information are imputed by sampling. Do the socialmixr figures show a single (random) iteration of this sampling?

**26 Response:** We thank the reviewer for this question. For the socialmixr figures, we do not need to conduct age imputation, as the participant axis corresponds exactly to the reported age groups.

**27 Comment:** Similarly, how are you selecting the final fitted parameters to generate your shown contact matrices from your posterior distribution?

**27 Response:** Thank you. We clarified in all figure legends that posterior median estimates of contact intensity matrices are shown.

**28 Comment:** Figure 4: is it possible to show the intensity for the top and middle rows at the same scale as the bottom row? E.g. by dividing the value for each cell for each integer age in the contact age group, and copying this value for each age in the participant age group? This also makes the comparison between age groups a bit fairer, as some are in 5y age bands and others are in 10y age bands.

**28 Response:** We thank the reviewer for this suggestion. We have edited Figure 4 as well as the supplemental figures accordingly.

**29 Comment:** Figure 5: how are the central estimates calculated? The median of posterior samples?

**29 Response:** We thank the reviewer for pointing this out. The central estimates are medians of the posterior samples. We have added explanations to every corresponding figure to clarify this.

**30 Comment:** Figure 5, 192: I agree with the attempted adjustment for reporting fatigue. However, it would be useful to understand how well your method is able to make this adjustment. Your rho parameter essentially rescales all contacts for people who have completed >1 survey. Could you compare the adjusted estimates in figure 5 to a model fit where all data is restricted to new participants?

**30 Response:** We thank the reviewer for this excellent suggestion. It is impossible to perform this experiment for all waves as most participants in waves 2, 3, and 5 are repeating participants. However, we compared the results of our final longitudinal model with a cross-sectional model fitted to data from wave 4 where all repeating participants are excluded from the data. We have included the results of our experiment as Supplemental Figure 16 and also added the following sentence to the text:

“Furthermore, we compared the marginal contact intensities of wave 4 given by the final longitudinal model, which adjusts for reporting fatigue with a cross-sectional model fitted on data from the same wave but with repeating participants excluded from the data (S16 Fig)”

**31 Comment:** Figure 7: The POLYMOD estimates seem very high. The mean contacts in the Mossong et al. paper are around 10-15 for most age groups, and similar for Germany. Could you double-check these? Have you included the estimated indirect contacts in the POLYMOD estimates? Because of different survey methodologies (and changes in behaviour), the differences between POLYMOD and COVIMOD are probably not only due to the pandemic, which should be accounted for when comparing the two surveys.

**31 Response:** Thank you for this comment. The main reason for the high contact rates for POLYMOD is due to the fact that we accounted for group contacts (indirect contacts) within our model, which was not done in the Mossong et al. paper. We clarified the legend of Figure 7 as follows:

“Dashed lines represent posterior median estimates from the pre-pandemic POLYMOD study and unlike the estimates reported in [1] include reported aggregate contacts.”

**32 Comment:** Line 484: This is figure 7.

**32 Response:** We thank the reviewer for pointing out this error in the numbering. We have addressed this issue in the updated manuscript.

**33 Comment:** Line 485: I don’t think you can conclude that there has been a sustained behavioural change compared to pre-pandemic values, as the two surveys are not directly comparable.

**33 Response:** We thank the reviewer for this comment. We have re-written this section in the Discussion for clarity as follows around line 491:

“In applying our model to the first five waves of the COVIMOD study, we gained insights into the age- and gender-specific social contact dynamics and their evolution over time at a resolution of 1-year age bands. We found relatively limited rebounds in contact intensities after the lifting of contact reduction measures, and that social contact intensities remained substantially below pre-pandemic values until the end of our study period in July 2020 [...]. These results are consistent with social contact survey data in England, Belgium, the Netherlands, and elsewhere.”

**34 Comment:** Line 528: I assume this is a computational limitation? Arguably, the contact patterns by age are much more important than those by/between gender. Combining the two genders may allow fitting to a larger number of survey waves.

**34 Response:** We thank the reviewer for this comment. It is a computational limitation, and combining contact intensity estimates across genders facilitates analyses over more survey waves. We will report on these as soon as possible.

We hope these revisions strengthen our manuscript and appropriately correct issues brought to our attention by the reviewers. We look forward to hearing from you and are prepared to make further revisions if needed.

Sincerely,

Shozen Dan, Oliver Ratmann (corresponding authors)

Imperial College London

Department of Mathematics
